# A novel contactless technique to measure water waves using a single photon avalanche diode detector array

**DOI:** 10.1098/rspa.2020.0457

**Published:** 2021-03

**Authors:** R. Zhang, S. Draycott, I. Gyongy, D. M. Ingram, I. Underwood

**Affiliations:** ^1^ School of Engineering, The University of Edinburgh, Edinburgh EH9 3DW, UK,; ^2^ School of Engineering, University of Manchester, Manchester M13 9PL, UK

**Keywords:** single photon avalanche diode, water wave measurement, specular reflection, optical profiling

## Abstract

Commonly deployed measurement systems for water waves are intrusive and measure a limited number of parameters. This results in difficulties in inferring detailed sea state information while additionally subjecting the system to environmental loading. Optical techniques offer a non-intrusive alternative, yet documented systems suffer a range of problems related to usability and performance. Here, we present experimental data obtained from a 256 × 256 Single Photon Avalanche Diode (SPAD) detector array used to measure water waves in a laboratory facility. 12 regular wave conditions are used to assess performance. Picosecond resolution time-of-flight measurements are obtained, without the use of dye, over an area of the water surface and processed to provide surface elevation data. The SPAD detector array is installed 0.487 m above the water surface and synchronized with a pulsed laser source with a wavelength of 532 nm and mean power <1 mW. Through analysis of the experimental results, and with the aid of an optical model, we demonstrate good performance up to a limiting steepness value, *ka*, of 0.11. Through this preliminary proof-of-concept study, we highlight the capability for SPAD-based systems to measure water waves within a given field-of-view simultaneously, while raising potential solutions for improving performance.

## 1. Background and Introduction

Optical techniques are an attractive approach for measuring water surface waves as they offer a non-intrusive approach which limits surface disturbances and the subsequent effect on both the wave measurement itself and other equipment/instruments. For ocean applications, this also avoids exposure of the instrument to ocean conditions thus reducing the effect of corrosion and wave loading [1]. However, optical systems are seldom used for wave measurement in ocean or laboratory settings due to a range of issues with performance and usability.

Optical techniques used to systematically measure water surface waves can be dated back to the first half of the twentieth century, with early attempts focusing on the use of stereo photography [2]. Although simple to set up and offering spatial measurements over large areas, the method can require manual processing and is historically computationally challenging [3,4]. Recent advances in computational power and advanced processing methodologies have overcome many of these issues (e.g. [5]). Of particular note is the open-source Wave Acquisition Stereo System (WASS) [6,7] which autonomously processes images to provide high quality processed surface elevations. However, issues persist with stereo photography related to the treatment of specular reflection, noise, quantization error and uncertainty of spatial position, making the application to high-fidelity laboratory experiments uncommon.

Other optical techniques have been developed based on exploiting light refracting through, and reflecting from, the water surface. Refraction-based methods effectively indirectly measure the surface slope (e.g. [8]). Recent refraction-based approaches offer good measurement performance. The methods presented in [9,10] are able to reconstruct complex wave fields with very high spatio-temporal resolution. There are some inherent limitations in terms of surface deformations/slope. However, the obvious shortcoming of refraction-based techniques is the requirement to submerge components, which offers installation challenges as well as resulting in a (potentially) intrusive measurement approach which itself is subject to wave loading.

Conversely, reflection-based methods excel in being non-intrusive and a number of experiments have been conducted using different optical systems. LIDAR has proven to be effective at measuring the water surface in both laboratory and ocean settings [11,12]. High spatio-temporal resolution can be achieved with good agreement to conventional wave gauges [11]. Issues with LIDAR are mostly related to the effect of the scanning (rotating mirror): spatial points are not time-synchronized and can have uneven spatial distribution for many LIDAR systems, and specular reflection limits its usable range due to the high angles of incidence for some mirror angles. Other effective approaches exist including three-dimensional particle image velocimetry (3D-PIV) [13] and particle tracer velocimetry (PTV) [14]. These methods, however, require tracers to be added, which significantly limits applicability.

Time-of-Flight (ToF) techniques have been recognized as a key approach for measuring distance between a sensor and an object of interest. They rely on emitting modulated or pulsed (typically infrared) light then measuring the time it takes to return to the sensor, after being reflected from the object. The approach enables real-time depth information to be obtained over an illuminated region and is thus a promising approach for the measurement of water surface waves. However, the reflectivity of the air/water interface is low (approx. 2% reflected [15]), and previous attempts using depth sensors with active illumination have shown that they typically require the addition of dye to the water [16]. Furthermore, techniques based on indirect ToF have difficulties dealing with multi-path returns [17], which is a problem for laboratory-based experiments where an additional return signal is expected from the basin floor.

Here, we present a water surface gravity wave detection system based on a pulsed solid-state laser (emitter) and an integrated array of CMOS-compatible single photon avalanche diode (SPAD) (sensors) operated under the ToF principle. SPAD detectors are a class of photodiode which can detect the arrival (and time of arrival) of a single photon. Due to their capability to achieve picosecond timing resolution, the devices have been used in a range of applications, including light-in-flight imaging [18], computational imaging (such as seeing around corners [19]), quantum imaging [20] and in biophotonics [21]. SPADs have also become a key technology for 3D time-of-flight imaging (or LIDAR), with SPAD-based ToF modules finding application in smartphones, robots and automotive LIDAR [22]. Traditionally realized as single-point detectors (requiring optical scanning to cover a field of view), with separate timing modules, recent developments have resulted in array format SPAD sensors with integrated photon timing and processing electronics [23]. These advances are now supporting advanced ToF imaging, including underwater [24] and at high speeds [25].

For the system presented herein, FPGA-based hardware controls the SPAD detector array and processes the resulting data to calculate the distance. The resulting system has several potential advantages over other techniques: picosecond resolution measurements can be obtained over an *area* of the water surface without the use of dye. In addition, a simple ToF-based data processing procedure is implemented which does not sacrifice the accuracy of measurement, deals with multi-path returns, and enables real-time depth measurements to be obtained over the entire SPAD detector array. Furthermore, due to the sensitivity of the SPAD, low powered ‘eye-safe’ lasers can be used.

In this paper, we present a proof-of-concept study on the performance of a 256 × 256 SPAD detector array for the measurement of water waves. It was not known prior to these experiments whether a SPAD detector could effectively capture a water surface, or indeed, a moving water surface in the form of waves. A series of wave conditions are generated at the FloWave Ocean Energy Research Facility (e.g. [26]), Edinburgh, UK and SPAD detector measurements are compared to those from a standard resistance-type wave gauge. In these tests, detectors in the array are combined into 4 × 4 groups to form 64 × 64 ‘macropixels’ under operation. The field of view of the camera is adjusted using a 3 mm/f2 lens placed in front of the SPAD detector array, providing a resulting macropixel size on the water surface of 0.7 cm. The SPAD detector array is synchronized with a pulsed laser source with a wavelength of 532 nm and the associated mean optical power is less than 1 mW. The laser and SPAD detector are installed with a 0.487 m vertical ‘stand-off’ distance and tested under 12 regular wave tests spanning a range of steepness with varied amplitude and frequency. A simplified optical model is developed and presented to better understand the results, and to quantify the performance in terms of wave steepness for the current laser-SPAD detector array configuration. The results are used to provide a critical assessment of the preliminary performance of the SPAD detector array for measuring waves along with suggesting potential improvements to expand the usable range of the sensor for wave measurement.

The remainder of the paper is laid out as follows. In §2, we describe the relevant theory, including the calculation of distance from time-of-flight measurements and the simplified optical model. In §3, the experimental set-up is detailed, along with the test conditions used. Results are presented in §4 including raw outputs, comparisons with wave gauge measurements and predicted outputs (from the optical model). Further discussion on performance and potential improvements are presented in §5 with concluding remarks offered in §6. An accompanying dataset, including wave gauge measurements, animations, and processed SPAD-depth data can be accessed at https://doi.org/10.7488/ds/2811.

## 2. Theory

### (a) Inferring distance from time-of-flight measurements

We used the technique of direct time-of-flight [27], whereby the target is illuminated with a pulsed laser source, and reflected photons are detected and timed using what is, in effect, an electronic stopwatch, synchronized with the laser. A histogram of photon arrival times is built using the time differences between the start provided by the laser and the stops provided by the SPAD detectors. By extracting the peak of the histogram, we can recover the time-of-flight *τ* of the laser pulse, which is directly proportional to the distance *d* to the target

**Figure 1.**
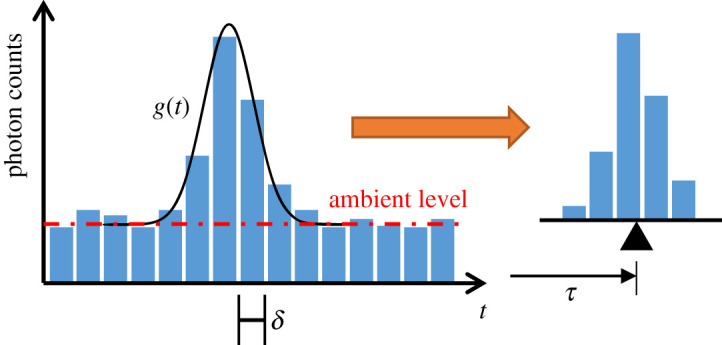
Time-of-flight histogram. (Online version in colour.)

2.1 $$d=\frac{c\tau }{2},$$

where *c* is the speed of light. Figure 1 shows a typical timing histogram, which can be approximated as a sampled Gaussian function with a vertical offset, arising from ambient photons (the underlying Gaussian profile *g*(*t*) resulting from the convolution of the laser pulse with the impulse response function of the ToF sensor). The bin width *δ* corresponds to the timing resolution of the system, and the photon count in each bin is subject to Poisson noise. A potential way to obtain the depth estimate, as encoded in the time position of the histogram peak, is via iterative curve fitting [28]. However, a simple centre-of-mass method (CMM) [29], as adopted here, leads to similar performance, while enabling real-time processing and depth visualization for an entire ToF sensor array. More specifically, the histogram is processed as follows. The median of the bins counts is subtracted from all bins to reduce the effect of background photons. Next, the bin with the maximum count is identified, and taking this bin, together with two neighbouring bins on either side, the centroid is computed. Scaling the value of the centroid as per equation (2.1) gives the depth estimate *d*

2.2 $$d=\frac{c\delta }{2}\left[\left\{\sum_{i=i_{\text{max}}-2}^{i_{\text{max}}+2}i(h_{i}-h_{\text{med}})\right\}/\sum_{i=i_{\text{max}}-2}^{i_{\text{max}}+2}(h_{i}-h_{\text{med}})\right],$$

where *h*_*i*_ (*i* = 1, …, *n*) are the histogram bins, *i*_max_ is the index of the bin with the maximum count and *h*_med_ is the median bin count.

We test the statistical significance of the histogram peak by comparing the maximum bin count with the threshold

2.3 $$h_{\text{thresh}}=h_{\text{med}}+4\sqrt{h_{\text{med}}}.$$

Recalling the Poisson statistics of the histogram bins, we note that each bin count has standard deviation equal to the square root of the underlying mean count. Thus the threshold in equation (2.3) requires the peak to be at least four standard deviations above the ambient photon level for it to be considered a ‘true’ peak with depth *d* estimated by equation (2.2). In the case that the peak falls below this threshold, *d* is taken as being undefined.

It is found that for a significant peak, provided the laser energy is spread over multiple (ideally 2–3 bins [30]), sub-bin precision can be obtained in the corresponding depth estimate, the standard deviation of *d* being approximately

2.4 $$\sigma_{d}=\frac{c}{2}\frac{\sigma }{N},$$

where *σ* is the standard deviation of *g*(*t*) and *N* is the (mean) number of signal photons within the peak.

### (b) Optical model

This section describes the basics of the light reflection from water surfaces, and uses this to develop an optical model able to predict when specular reflection reaches different macropixels (4 × 4 pixels) as a function of the instantaneous surface slope.

**Figure 2.**
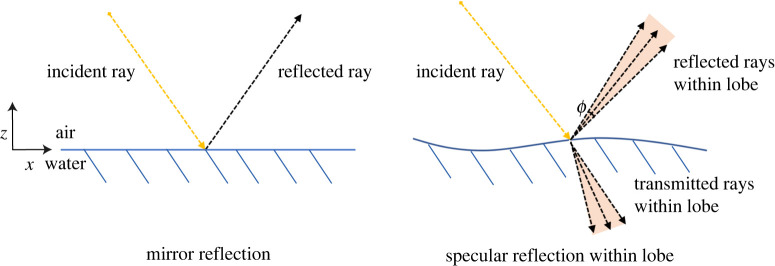
Ideal mirror reflection and specular reflection lobe. (Online version in colour.)

A completely flat still water surface can be seen as a specular reflector, where the angle of the reflected ray is equal to the incident angle with respect to the local reflected surface normal. However, when the surface is disturbed by wind or waves, the water surface is no longer a perfect mirror-like plane. In this case, the surface is more like an array of adjacent micro-mirrors with similar but not identical orientation, which will result in a spread of directions around that which would be expected from a perfect mirror-like surface. Importantly, only around 2% of the radiation will get reflected, with the remaining 98% being transmitted [15]. These factors are of critical importance in understanding which conditions can be captured by the SPAD detector array. Here, we adopt the simplified Phong model [31] which is commonly used in computer graphics. This approach essentially models reflections as a weighted sum of diffuse and specular components. A key parameter for this model is the specular ‘lobe’ which captures the angles which bound the reflected components around the central mirror-like direction. This is shown in figure 2, along with a representation of the reflected and transmitted rays. The shape and angle contained within the lobe changes depending on the position of the light source and the optical properties of the surface of the illuminated material. For water waves, as the slope changes so does the centre of the lobe and hence reflection direction, however as the medium is unchanged the angle contained within the lobe itself is approximately constant.

**Figure 3.**
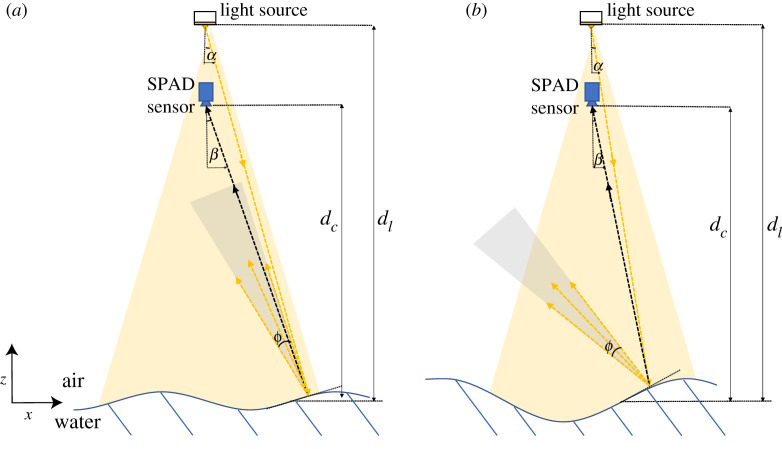
Diagram depicting the specular reflection cone and implications for wave measurement [Not to scale]. Shows (*a*) a low steepness wave condition where the light ray can always reach the SPAD detector; and (*b*) a high steepness wave condition where the reflection cone cannot always reach the SPAD detector. (Online version in colour.)

Figure 3 illustrates the implications of this reflection lobe using two scenarios. The first is demonstrated for the case where the lobe is wide enough, and steepness low enough, for reflected photons to always reach the detector. The second is a higher steepness wave case with the same lobe (same material) where at certain instances no light reflects into the detector. (Note that the figure is not in proportion to the full-scale setup.) In our experiments, the light source and the camera are oriented on the vertical axis to have the same *x* position, with waves propagating along the positive *x*-axis. The macropixel size (*ps*) is calculated to be 0.7 cm at the still water surface. The relative position of an individual macropixel *i* can therefore be calculated using *i* · *ps*. We label the macropixel directly beneath the SPAD detector array (*x* = 0) to be *i* = 0. The wave surface is only visible when one of the light rays within the reflection lobe (shown in grey) reaches the SPAD detectors. To estimate the visibility of each macropixel across the range of slopes imposed by the wave, we need to consider the instantaneous gradient of the surface (d*η*/d*x*), the angle of incident light ray to the macropixel (*α*), the angle between the SPAD detector and macropixel (*β*), and the angle of the reflection lobe (*ϕ*).

Based on figure 3, the following analysis derives the limiting surface slope, and hence wave steepness *ka* (where *k* is the wavenumber [m^−1^] and *a* is the wave amplitude [m]) for a given macropixel to obtain meaningful data. Note that the wavelength *λ* = 2*π*/*k*. *k* is related to the angular frequency, *ω* [rad s^−1^], through the linear dispersion relation $w=\sqrt{gk\tanh kh}$, where *h* is the water depth and *g* is acceleration due to gravity.

Assuming linear wave theory, the surface elevation, *η*, for a sinusoidal surface gravity wave can be expressed as

2.5 η(t)=asin⁡(kx−ωt)=asin⁡(k⋅i⋅ps−ωt)

The instantaneous surface slope can be expressed as

2.6 $$\frac{\text{d}\eta }{\text{d}x}(t)=ka\cos(k\cdot i\cdot ps-\omega t).$$

Denoting the distance between the light source and still water surface as *d*_*l*_, the distance between the SPAD detector and still water surface *d*_*c*_ and the lateral distance between camera and light source *d*_*lc*_ (0 for our set-up) it can be concluded that

2.7 $$\alpha =\tan^{-1}\left(\frac{i\cdot ps-d_{lc}}{d_{l}-\eta (t)}\right)$$

and

2.8 $$\beta =\tan^{-1}\left(\frac{i\cdot ps}{d_{c}-\eta (t)}\right).$$

Hence the limits of water surface gradients (d*η*/d*x*) where specular reflection makes it to the SPAD detector can be calculated using

2.9 $${\left(\frac{\text{d}\eta }{\text{d}x}\right)}_{\text{lim}}=\tan\left(\left[-\frac{\phi }{4},\frac{\phi }{4}\right]+\frac{\alpha }{2}+\frac{\beta }{2}\right).$$

From equation (2.6), it can be inferred that the maximum value of d*η*/d*x* at any location (macropixel) will be equal to *ka* and hence if a macropixel *i* is to obtain 100% of the data throughout a wave period the maximum value that *ka* can take is

2.10 $${\left(ka\right)}_{\text{max}}=\min\left\{\left|\tan\left(\frac{\phi }{4}+{\frac{\alpha }{2}|}_{\eta =0}+{\frac{\beta }{2}|}_{\eta =0}\right)\right|,\left|\tan\left(-\frac{\phi }{4}+{\frac{\alpha }{2}|}_{\eta =0}+{\frac{\beta }{2}|}_{\eta =0}\right)\right|\right\}$$

noting that when d*η*/d*x* is maximum and equal to *ka* that *η* = 0. *α* and *β* are both a function of the macropixel position *i* and hence the limiting steepness is pixel-dependent. At instances where d*η*/d*x* exceed the values resulting from equation (2.9) the specular reflection can not reach the camera and the signal will be regarded as NaN (Not a Number). The distance from the laser source and camera to the still water surface are fixed, hence the factors that dominate the performance are the macropixel position, along with the amplitude and frequency (and hence *k*) of the wave. §4 shows the experiment results and compares them to the prediction of this optical model.

## 3. Experimental configuration and test plan

### (a) Experimental set-up

All experiments presented in this manuscript were carried out in the FloWave Ocean Energy Research Facility (FloWave) based at the University of Edinburgh. FloWave is a circular combined wave-current facility with a diameter of 25 m and a nominal water depth of 2 m. A range of regular (monochromatic) wave tests were generated in FloWave to gain a fundamental initial understanding of the performance (see §3c for conditions used).

**Figure 4.**
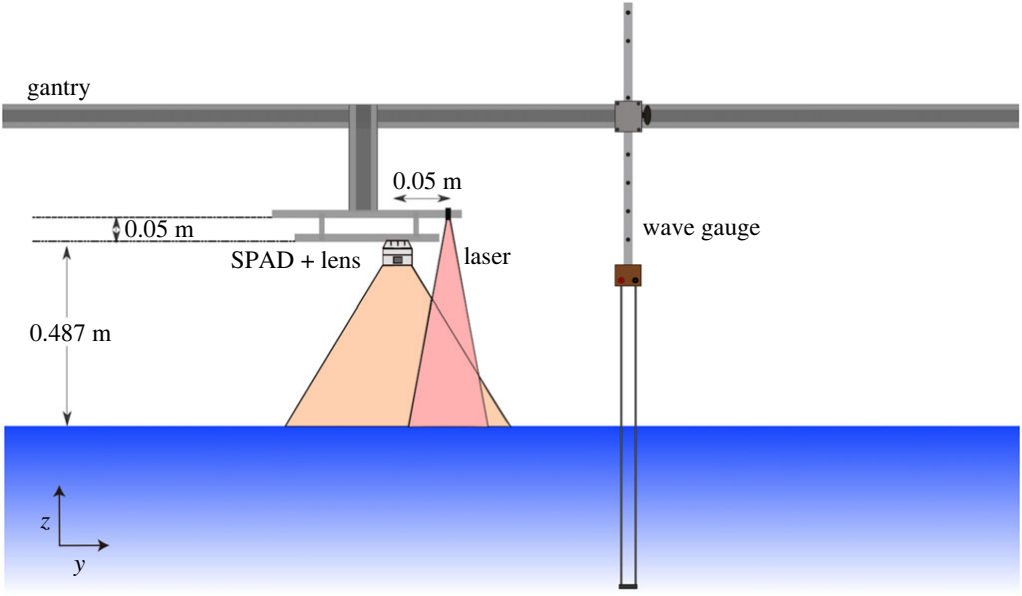
Diagram of test set-up [Not to scale]. SPAD detector and lens shown including indicative measurement and illumination regions. Wave gauge shown next to the SPAD detector measurement area, noting that wave travel (and the positive *x*-direction) was into the page. (Online version in colour.)

In addition to the SPAD detector array-laser set-up (§3b), a single resistance-type wave gauge was installed (manufactured by Edinburgh Designs Ltd.) to enable comparison and validation of the outputs from the SPAD detector array. This was mounted very close to the SPAD detector array measurement area, and in the same *x* position (waves travel is along the positive *x* axis). This ensures any unwanted wave reflections have the same phase relationship with the incident waves and hence ensures the same wave amplitudes are observed in both measurement systems. The SPAD detector is placed with a mean stand-off distance of 0.487 m, aimed at minimizing parallax effects (and water splashing), while maximizing photon returns. The test set-up, including the SPAD detector array, laser source and wave gauge is depicted in figure 4.

### (b) Single photon avalanche diode detector and laser configuration

The SPAD detector used here is a 256 × 256 array with 9.2 *μ*m pitch, featuring a 3D stacked architecture with separate detector and photon processing tiers [32]. The stacked structure results in a high fill factor of 51% (representing the active—or photosensitive—area of each pixel as a percentage of the overall pixel area) and enables on-chip histogramming functionality. In this mode, pixels are combined in 4 × 4 groups to form 64 × 64 ‘macropixels’, each of which generates a 16-bin histogram of the time of arrival of detected photons (with 14-bit counts in each bin). The bin width was configured here to be 700 ps. The sensor also offers a 14-bit photon counting (or intensity imaging) mode, at the full 256 × 256 resolution of the array, which was used here to set the focus of the 3 mm/f2 objective in front of the SPAD detector. A hardware issue in the prototype sensor currently restrict readout to approximately half the array (resulting in a usable 64 × 27 macropixel array), and to 15 out of the 16 histogram bins.

The sensor is attached to a custom-designed camera board also comprising an FPGA integration module (Opal Kelly XEM6310), which controls the sensor, and relays its output to a PC via a USB 3.0 link. On the PC, a Matlab-based software interface orchestrates the acquisition of, and decodes the data frames into 3D (depth) images.

Exposure time was set to 100 ms, leading to a frame rate of approximately 10 frames per second (fps), with each frame consisting of a 64 × 27 × 15 data cube of histogram data, which is then converted to a 64 × 27 depth frame using centre-of-mass processing as detailed in §2a. For all of the tests, the SPAD detector array is configured to record 300 depth images, equating to around 30 s of data, per test. All tests were carried out in ambient light conditions with natural light present from windows and laboratory operating lights turned on.

The camera was configured to trigger, with 60 MHz repetition rate, a pulsed green laser source (Picoquant LDH with 532 nm laser head; the photon detection probability of the SPAD detector at this wavelength is greater than 10%). A green laser source was used due to higher sensitivity of the SPAD detector array at the blue end of the visible spectrum. The light from the laser was coupled into a multi-mode optical fibre, emerging at the other end as a circular illumination spot with average optical power of less than 1 mW. Due to the relatively low laser power, the fibre was positioned so as to illuminate only a sub-region in the field of view of the SPAD detector array, corresponding to around 25 × 15 macropixels (the remaining macropixels hence detecting ambient photons only, with no distance information).

At the beginning of the experiment, a zero calibration (or offset correction) frame was captured by imaging the still water surface, which was then subtracted from subsequent depth frames. Gain calibration was performed empirically and post-experiment by reconciling the peak-to-peak amplitude of the SPAD measurements with the corresponding wave gauge data one of the test cases (Test 6 in table 1, a low steepness condition was well captured by the SPAD detector array). Based on this approach, the empirical gain value was calculated to be 1.67, and was subsequently applied to all macropixels for all test cases.

**Table 1.** Test conditions

ref.	target (actual) *f* [Hz]	target amp *a* [m]	estimated steepness *ka*	test type
1	0.7 (0.688)	0.02	0.0395	amplitude sweep
2	0.7 (0.688)	0.04	0.079	amplitude sweep
3	0.7 (0.688)	0.06	0.118	amplitude sweep
4	0.7 (0.688)	0.08	0.158	amplitude sweep
5	0.7 (0.688)	0.1	0.197	amplitude sweep
6	0.3 (0.313)	0.06	0.0291	frequency sweep
7	0.4 (0.406)	0.06	0.0432	frequency sweep
8	0.5 (0.5)	0.06	0.0623	frequency sweep
9	0.6 (0.594)	0.06	0.0875	frequency sweep
10	0.8 (0.813)	0.06	0.155	frequency sweep
11	0.9 (0.906)	0.06	0.196	frequency sweep
12	1 (1)	0.06	0.242	frequency sweep

### (c) Test plan

The input wave conditions are defined in table 1. They consist of a set of constant frequency tests of varying amplitude, *a* (refs. 1–5) and a set of tests with constant amplitude and varying frequency *f* (refs. 6–12); wave period *T* = 1/*f*. This creates a range of wave steepness values as detailed in the table. There are comparable values of steepness from both the amplitude sweep and frequency sweep tests, enabling isolation of whether performance is dominated by frequency, amplitude or steepness.

In the table, the target frequency and actual frequency differ for most tests. This is a result of the repeat time, *T*_*r*_ used in the tests of 32 s, meaning that the tank generation frequencies have to be multiples of 1/*T*_*r*_, i.e. *N*/32. Note that a 0.7 Hz test for the frequency sweep was not included as would be identical to test 3. Amplitude values presented are target (input) amplitudes with measured amplitudes expected to differ slightly as a function of frequency [33]. Steepness values shown are those calculated from the wavenumbers associated with the known (actual) frequencies along with the target amplitudes.

## 4. Results

### (a) Distance sweep of solid target

**Figure 5.**
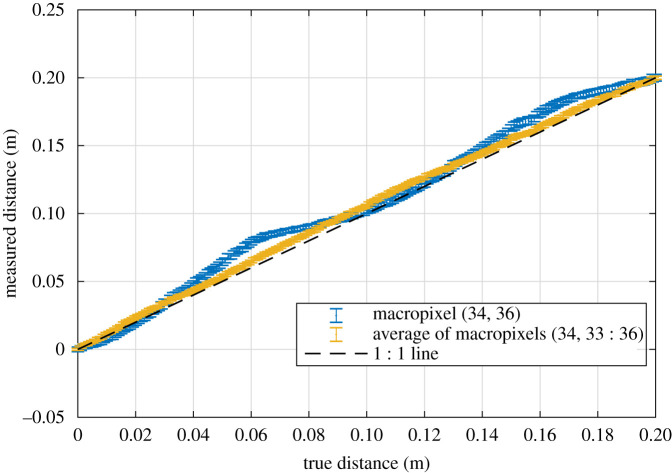
Results of the distance sweep experiment, showingthe mean depth value for a single macropixel (blue) and the average of four macropixels (yellow) across 100 repetitions at each distance. Errorbars represent the standard deviation over the 100 repetitions. (Online version in colour.)

To ascertain the accuracy and precision achievable using the SPAD sensor and laser source in this study, a test was carried out in controlled conditions. The imaging system was pointed to a flat cardboard target on a translation stage, which was then moved, away from the sensor, in 1 mm increments over a 0.2 m distance, starting from a 0.5 m distance. To ensure, the conditions were representative of the tests in the tank, the laser power was adjusted such that macropixels observing the centre of the illuminated spot would capture a similar number (approx. 500) signal photons in each 100 ms exposure at a 0.5 m distance. 100 frames were captured at each distance step so that mean and standard deviation values could be calculated for the depth measurements of each macropixel in the SPAD. The results for the mean measurements are shown in figure 5, both in terms of a single macropixel (blue markers) and the average of the measurements from four neighbouring macropixels (yellow markers). In both cases, the measured distance has been calibrated so as to match the true distance at 0.5 and 0.7 m. The measurement of the single macropixel is found to be considerably nonlinear, which is assumed to be largely due the variability in the effective histogram bin width (which is as much as ±5%. [32]). With the nonlinearity uncorrected, the distance error (bias) is up to 20 mm within the range considered. It is interesting to note that by averaging pixels, the measurement becomes more linear, the maximum error reducing to below 10 mm. The standard deviation of depth estimates (for single macropixels) was measured to be 2.2 mm at 0.7 m distance, reducing to 1.6 mm at 0.5 m.

### (b) Raw single photon avalanche diode detector detector array outputs

**Figure 6.**
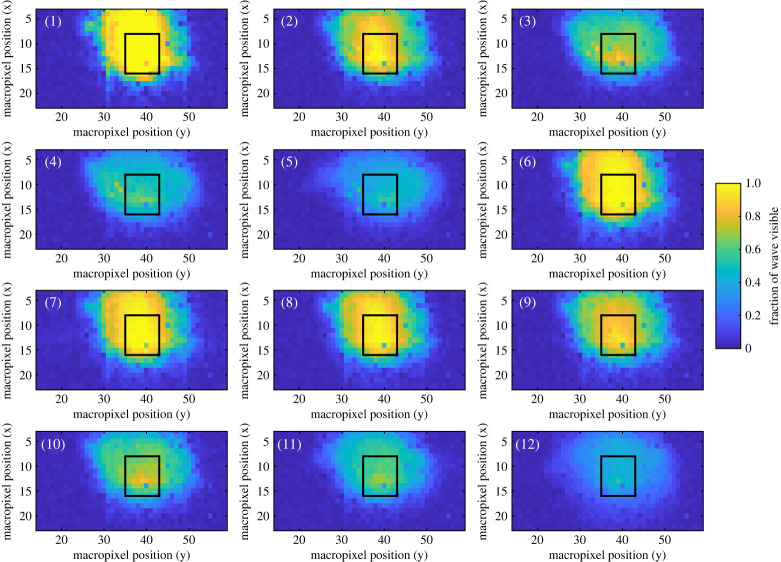
Comparison of the fraction of time the surface is visible for different macropixels, and for tests 1 to 12 (table 1). The black rectangle denotes the area subsequently used for demonstrating post-processing techniques in figures 12 and 13. (Online version in colour.)

This section presents the raw histogram and depth data obtained from the SPAD detector array. Due to relatively low laser power used, along with the fibre-optics used and distance to the water surface, only a limited surface region was sufficiently illuminated; with the remaining area only detecting ambient uncorrelated information. Subsequently, the area that receives useful reflections in this experiment is around 25 × 15 macropixels. This also varies as a result of macropixel position and instantaneous surface slope as explained in §2b. To experimentally assess this, figure 6 display the fraction of time a useful signal is obtained for all macropixels and for each of the 12 tests. It is clear that, in relatively low steepness tests (1, 2 and 6–9), a high proportion of data is collected for most of this illuminated region. As the steepness increases (amplitude tests 1→5, frequency tests 6→12) the amount of useful data reduces significantly. None of the macropixels in tests 3–6 and 10–12 has a high proportion of data collected. This result is expected from §2b: with a single SPAD detector array and single light source the amount of useful data will decrease with wave steepness. Potential improvements to solve this problem are discussed in §5d.

**Figure 7.**
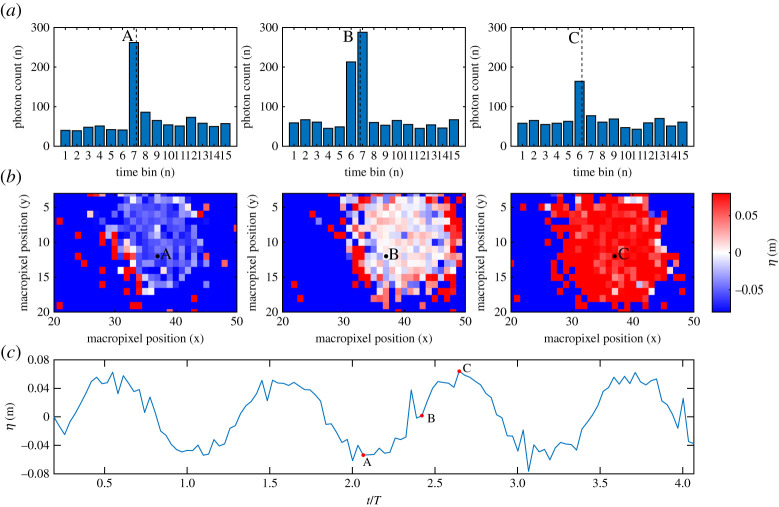
SPAD detector outputs for a macropixel beneath the SPAD detector and laser (*i* = 0) for test 6: *a* = 0.06 m, *f* = 0.3 Hz. (*c*) Time series for macropixel *i* = 0. (*b*) Depth surfaces for all macropixels at times corresponding to A, B and C. (*a*) Histograms corresponding to macropixel *i* = 0 for times A, B and C. (Online version in colour.)

**Figure 8.**
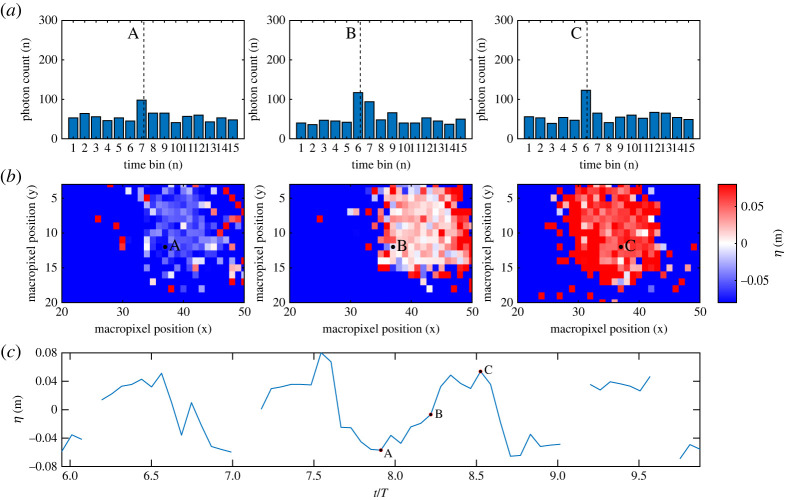
SPAD detector outputs for a macropixel beneath the SPAD detector and laser (*i* = 0) for test 9: *a* = 0.06 m, *f* = 0.6 Hz. (*c*) Time series for macropixel *i* = 0. (*b*) Depth surfaces for all macropixels at times corresponding to A, B and C. (*a*) Histograms corresponding to macropixel *i* = 0 for times A, B and C. (Online version in colour.)

Raw outputs from the SPAD detector array for two tests, 6 (*a* = 0.06 m, *f* = 0.3 Hz) and 9 (*a* = 0.06 m, *f* = 0.6 Hz), are presented in figures 7 and 8, respectively. These figures show examples of the histograms, depth surfaces and individual pixel time-histories obtained from the SPAD detector array. Common points (in time and/or space) are indicated by A, B and C. Point A is a specific instance for a pixel beneath the laser and SPAD detector (*i*= 0, *x* = 0 mm) corresponding roughly to the trough of the wave. C is the same for the crest, and B is the same for a point of maximum steepness (d*η*/d*x* ≈ *ka*). The centroid resulting from the CMM is also labelled as a dashed line on the histograms.

Assessing first the histograms in figure 7, it is clear that the dominant bin for point A (trough) is 7, whereas for point C (crest) the dominant bin is 6 as the photons have travelled a shorter distance. For point B (max slope), the photon energy is split between bins 6 and 7. The surfaces in the middle row show the corresponding surface elevation values for all macropixels at points A, B and C. This demonstrates that a large number of macropixels are capturing the elevation effectively. The bottom plot shows the time-series extracted for the highlighted macropixel, over four wave cycles. It is evident that the surface elevation is captured reasonably well and with no data dropouts. Small ‘spikes’ are evident which are attributed largely to the low photon counts and hence resulting influence of uncorrelated photons on the CMM calculation.

Figure 8 shows equivalent results for a higher steepness condition with broadly similar outputs. For this test, however, a number of key differences are apparent. It is clear that the chosen macropixel does not always obtain meaningful data, highlighted by the data drop outs in the presented time series. Assessing the surfaces the reason for this becomes apparent: the range of macropixels obtaining meaningful data ‘moves’ with the wave phase (as predicted by §2b). For point B shown, the chosen macropixel is only just on the edge of the macropixels which are within the reflection cone, whereas for other similar points where d*η*/d*x* ≈ *ka* this macropixel is outside this range. The reason why some half-cycles are fully captured and some are not is dependent on the specific sampling time relative to the instantaneous surface slope (and potentially slight differences in the slopes of consecutive waves). For the reader’s interest, 12 videos are provided at https://doi.org/10.7488/ds/2811, corresponding to the 12 regular wave test cases. Each of these videos display processed depth data for all macropixels over the full 300 frames, along with accompanying histograms for macropixel *i* = 0.

### (c) Single photon avalanche diode detector detector performance and comparisonto optical model

This section compares the ability of the SPAD detector array to track the water surface for given wave conditions against that predicted by the optical model described in §2b. As evidenced in §2b, the expected performance is highly dependent on the specific macropixel position and the slope of the instantaneous water surface corresponding to this location. At certain instantaneous slopes, a given macropixel may not receive the reflected photons emitted from the laser source, resulting in poor signal strength and the allocation of a NaN (Not a Number) to the measurement for that macropixel and time-stamp. Hence to evaluate the performance of the SPAD detector array in different wave conditions, and the predictive capability of the model, the fraction of wave visible (usable data) can be assessed.

**Figure 9.**
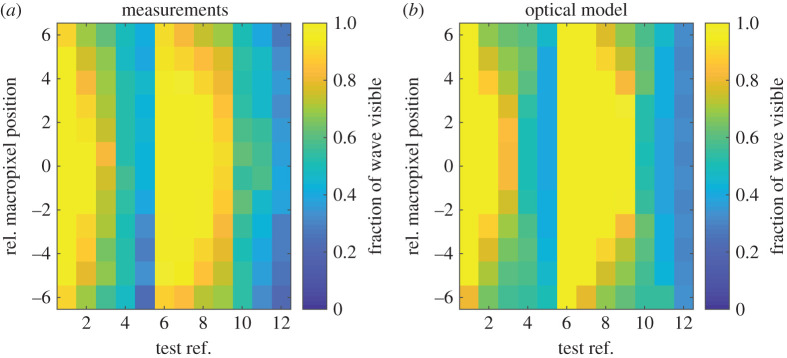
Fraction of wave surface visible as a function of macropixel position for each of the 12 tests. Shown for (*a*) measurements and (*b*) the optical model. Note that the position shown on the *y*-axis is positive in the direction of wave propagation. (Online version in colour.)

For the following comparisons, the value of *ϕ* was chosen to be 25^°^, based on agreement with the experimental results. Using equation (2.10) with *ϕ* = 25, the limiting *ka* value for a macropixel beneath the laser and SPAD (*α* = *β* = 0) is expected to be 0.1095. Figure 9 shows the measured, and predicted, fraction of wave visible as a function of macropixel position and test number. In this figure, macropixel position 0 corresponds to a central macropixel at *x* ≈ 0 mm, closest to being directly beneath the laser and SPAD detector array (relative to wave propagation direction). Relative macropixel position 6 refers to measurements taken 6 macropixels away from the central pixel in the wave propagation direction (*x* ≈ 42 mm), with −6 referring to a position 6 macropixels in the negative *x*-direction (*x* ≈ −42 mm). Assessing figure 9, it is clear that the model qualitatively predicts the performance of the SPAD detector array well; capturing the sensitivity to macropixel position and wave parameters. Noting that tests 1–5 increase in amplitude for a constant frequency, while tests 6–12 increase in frequency with a constant amplitude, it is observed that increasing the steepness serves to reduce the amount of usable data. This increases the proportion of time where the surface slope is large: resulting in no light path from the laser to the SPAD detectors making up the specific macropixel.

**Figure 10.**
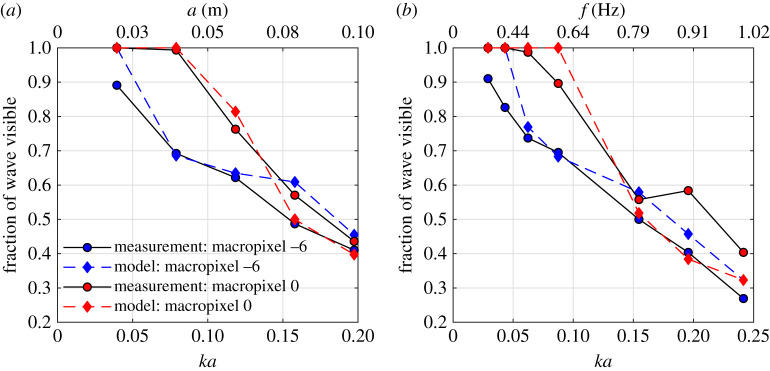
Fraction of expected (model) and measured wave surface visible for two relative macropixel positions (0 and −6) as a function of wave steepness. Shown for (*a*) amplitude sweep tests with constant target frequency of 0.7 Hz and (*b*) frequency sweep tests with constant target amplitude of 0.06 m. (Online version in colour.)

To further assess the sensitivity to steepness, and the model predictive performance, figure 10 is presented. This figure shows the fraction of wave visible against the wave steepness, *ka*, for both the amplitude (a) and frequency (b) sweep tests. Both measured and predicted values are shown for relative macropixel positions 0 (high proportion of data) and −6 (low proportion of data). It is clear that the wave steepness is, as expected, the key parameter determining the amount of usable data for a given macropixel. For both macropixels assessed, and for the frequency and amplitude sweeps, the fraction of wave visible reduces significantly with increasing *ka*. Similar performance is observed for tests of equivalent *ka* values, yet with different amplitudes and frequencies. The optical model predicts the fraction of useful data acceptably well for both macropixels and for all steepness’, suggesting it may be applied outwith the measurement range and to assess the expected performance of alternative configurations.

For a central macropixel (0), the predicted limiting steepness (*ka*) is about 0.11 (from equation (2.10)) for the current configuration to obtain 100% meaningful data, which agrees well with the experiments (although only up to 0.09 tested). This confirms that, as described in §2b, for a single laser–single SPAD detector array configuration there is an inherent limit imposed by the surface slope. Additional illumination sources and/or detectors will be required to overcome this, which is discussed further in §5d.

### (d) Comparison to wave gauge measurements

This section compares measurements from the SPAD detector array with those from the wave gauge. Two macropixels are chosen for comparison. One in a central position where there is mostly good data (denoted macropixel position 0 in figure 9), and one is 6 macropixels away in the negative *x* direction where the data becomes more sparse (denoted macropixel position −6). These are the same macropixels as presented in figure 10.

**Figure 11.**
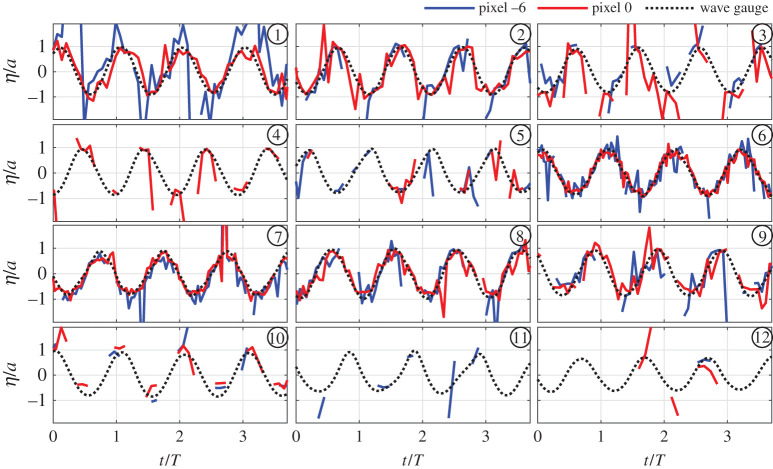
SPAD detector array measurements for two macropixels compared to wave gauge measurements. Shown for tests 1 to 12 defined in table 1. (Online version in colour.)

As the wave gauge and the SPAD detector array measurement system are not time-synchronized, to enable a better visual comparison in the time-domain the signals were aligned by finding the time-lag which maximizes the cross-correlation value between the SPAD-extracted signals and the wave gauge. The time-aligned wave gauge and SPAD detector array measurements for the two example macropixels are shown for the 12 tests in figure 11. Four wave periods are shown for each test, and are normalized by the target wave amplitudes and generated periods.

It is evident from figure 11 that, for the central macropixel (0), good agreement with the wave gauge data is obtained for the six lowest steepness tests (1, 2 and 6–9). Data for waves with *ka* values above around 0.09 is very sparse and consists of large errors. This supports findings from §4c that this may be close to the limiting steepness for capturing specular reflections with this set-up. For relative macropixel position −6, reasonable agreement is found only for test 6 with the lowest steepness. For all other tests, the data for macropixel −6 are sparse and/or has poor agreement with the wave gauge measurements. As expected a larger amount of data is collected for macropixel −6 for the lower steepness conditions, but there is still poor agreement with the wave gauges during these periods of measurement. This demonstrates that the fraction of wave visible, as assessed in §4c, is not a perfect measure of performance. There are instances where data is not discarded, yet the data collected is not meaningful, and hence careful consideration must therefore be given to which macropixels are used for subsequent analysis.

**Figure 12.**
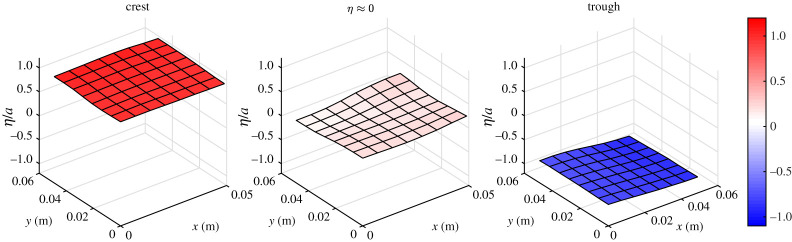
Example of the post-processed depth surfaces for a chosen 8 × 8 macropixel region. Shown for test 8 (*f* = 0.5 Hz, *a* = 0.06 m) at a moment corresponding to a crest, trough and instant where *η* ≈ 0. (Online version in colour.)

**Figure 13.**
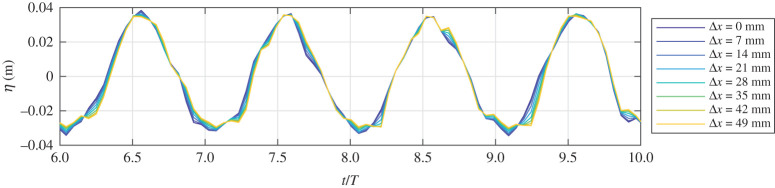
Example time series from post-processed depth surfaces. Eight macropixels in the direction of wave propagation shown for test 8: *f* = 0.5 Hz, *a* = 0.06 m. (Online version in colour.)

Even for low steepness conditions, there is some notable noise in all of the measurements, but it is clear that post-processing techniques will be able to reliably retrieve the true surface. A low-pass filter can be used to remove high-frequency noise, and a de-spiking algorithms can readily remove spikes. Example post-processed surface elevations are shown in figures 12 and 13 for test 8, using a low-pass filter set to 4 Hz and the histogram de-spiking method of [34]. Figure 12 shows an example 8 × 8 macropixel surface (corresponding to black rectangle in figure 6) for times associated with a crest, trough and points of maximum steepness, whereas figure 13 shows time histories for eight adjacent macropixels in the direction of wave travel. It is clear that the spikes and high-frequency noise have largely been removed and the signals much improved.

## 5. Discussion

### (a) Expected performance in irregular wave conditions

The results herein show the SPAD detector array measurement performance for regular wave conditions, highlighting an inherent limit for *ka* of about 0.11 for a central macropixel to obtain meaningful data throughout an entire wave cycle. Many tests in wave tanks, like real-world ocean conditions, are irregular, comprising a range of amplitudes and wavenumbers and consequently surface slopes. This results in more complex limiting conditions for irregular sea states.

**Figure 14.**
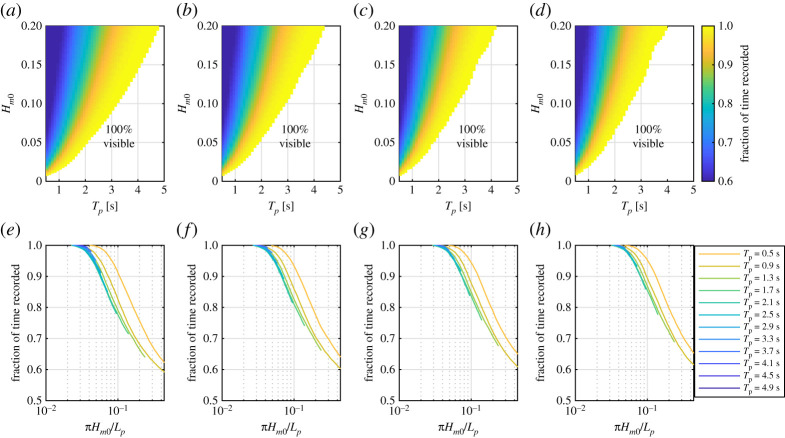
Estimated performance for a central macropixel in irregular conditions. Panels (*a*–*d*) correspond to *γ* values of 1, 3, 5 and 7, respectively, and depict the region where 100% measurement is expected along with the fraction of time the surface is recorded as a function of *H*_*m*0_ and *T*_*p*_. Panels (*e*–*h*) show the expected fraction of time where the surface is visible as a function of *πH*_*m*0_/*L*_*p*_ for *γ* values of 1, 3, 5 and 7, respectively. Each line corresponds to estimates associated with increasing *H*_*m*0_ for a given *T*_*p*_ and *γ*. (Online version in colour.)

Figure 14 shows the expected performance for a central macropixel in irregular conditions from the optical model presented in §3b. The conditions modelled correspond to the typical range generated in FloWave. JONSWAP spectra [35] are used to define the input energy density spectra, *S*(*f*), for a range of significant wave height (*H*_*m*0_), peak period (*T*_*p*_) and spectral bandwidth (*γ*), values. Surface gradients are linearly simulated using a sum over frequency components, *n*

5.1 $$\frac{\text{d}\eta }{\text{d}x}(t)=\sum_{n=1}^{Nf}k_{n}a_{n}cos(k_{n}\cdot i\cdot ps-\omega_{n}t+\phi_{n}),$$

where $a(f)=\sqrt{2S(f)\Delta f}$ and Δ*f* is the chosen frequency resolution. *w*(*f*) and *k*(*f*) are related through the linear dispersion relation and *ϕ*(*f*) is a randomly generated phase which is uniformly distributed between 0 and 2*π*. *i* is set to 0 in this example to simulate a central macropixel and *N*_*f*_ is the total number of frequencies used for the simulation.

Figure 14 shows the region where 100% of the surface elevation will be captured as a function of *H*_*m*0_ and *T*_*p*_ (panels *a*–*d*), and as a function of a spectral steepness parameter (panels *e*–*h*) defined as *πH*_*m*0_/*L*_*p*_, where *L*_*p*_ is the wavelength associated with the peak period. The estimated fraction of the surface which is visible beyond this limiting value is also presented.

Assessing panels (*a*–*d*), it is clear that *H*_*m*0_ and *T*_*p*_ dominate the limiting cases through defining the dominant amplitudes and wavelengths present which contribute to surface slope. *γ* plays a minor role in the definition of the limiting conditions, through specification of the relative magnitudes of higher frequencies with associated higher wavenumbers. Lower values of *γ* define a more broad-banded frequency spectrum, which is associated with lower limiting conditions due to the increased magnitude of the higher wavenumber components. Assessing panels (*f* –*h*), it is evident that the conditions which can be 100% measured by a central macropixel have significantly lower spectral steepness than 0.11, due to instances of higher surface slopes present in the irregular conditions. The limiting value of *πH*_*m*0_/*L*_*p*_ is closer to 0.05, but varies as a function of *T*_*p*_ and *γ*. The dependency on *T*_*p*_ is a result of the local gradient of the dispersion relation around the peak wavenumber/wavelength.

Figure 14 demonstrates that the limiting spectral steepness for irregular conditions is lower than the limiting *ka* value for regular wave conditions. Although the optical model predicts many conditions can be effectively measured, the limiting wave parameters are well below those which are routinely used in experimental tests, particularly for lower *T*_*p*_ values. Ways to improve/remove this limitation are discussed further in §5d.

### (b) Single photon avalanche diode detector detector-laser performance

Prior to this preliminary performance assessment of a SPAD detector array for the measurement of surface gravity waves, it was not known whether any meaningful data would be obtained. The experiments prove that a SPAD detector array-laser configuration can be used to obtain meaningful measurements of water waves, yet there is an inherent limit for measurement depending on the wave steepness and the macropixel position. A limiting *ka* value was estimated to be 0.11 for a central macropixel to measure the entire wave profile, which is then lower for all other macropixels. Ocean waves have a fundamental breaking limit of *ka* ≈ 0.44 and hence this is a significant limitation for measurement in either laboratory or ocean environments. These limits, however, do not reflect the SPAD detector array performance itself, and instead reflect the limitations of the configuration (discussed further in §5d).

The actual performance of the sensor-laser combination is very promising, yet it is noted in figures 7, 8 and 11 that there is a significant amount of temporal ‘noise’ in the signals. This is predominantly because of the low photon count resulting from the low powered laser (noting equation (2.4)). The fluctuating number of background photons counted in dominant or neighbouring bins is therefore a relatively large proportion of the peak and randomly affects the inferred depth value. Increasing the intensity of the laser, and adding a optical band-pass filter to the sensor (centred on the laser wavelength), should therefore resolve these issues. There is also a known glitching issue with the sensor itself (affecting around 1% of frames) which also causes spurious peaks to be recorded which can also be readily resolved in future iterations. If these changes are implemented then the signals below the limiting steepness should be much cleaner and require less post-processing (or none).

### (c) Optical model

In §2b, a simplified optical model was presented and used to understand the expected performance of the SPAD detector array in the current configuration for different wave conditions. The comparison with the measurements in §4c demonstrated that the model, with the chosen value of *ϕ*, can satisfactorily predict the proportion of data that will be obtained by each macropixel in the SPAD detector array. Assessing figures 9 and 10 in conjunction with figure 11, it is clear that when the model predicts a macropixel will collect 100% of the wave profile, that the surface elevation values are reliable. For any macropixel-condition combination where it predicts there will be some drop-outs the data appears poor, yet the fraction of data expected does not necessarily reflect the quality of the data that has been collected. Given this assessment, it appears that the optical model can be used as an effective indicator of performance in a binary sense. If assessing prospective performance in other conditions or for other configurations (see §5d) then it is suggested that 100% data collected should be considered as acceptable and anything lower as unusable.

### (d) Future improvements

**Figure 15.**
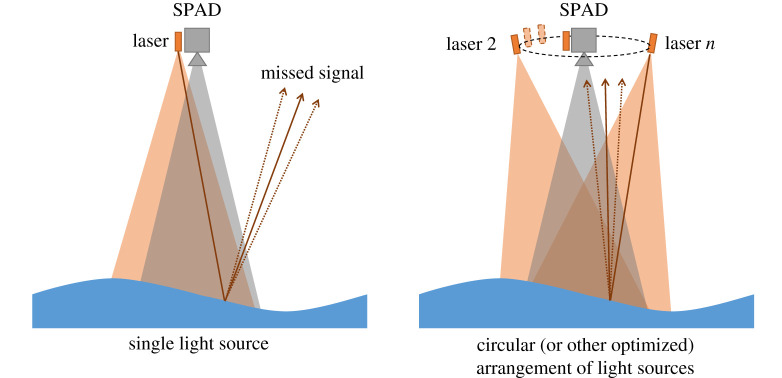
A potential multi-laser arrangement to overcome the wave steepness limit. (Online version in colour.)

Assessing the optical model outlined in §2b, it is evident that limits arise due to a combination of the instantaneous surface slope and the angle of incidence of the laser ray(s). No signal will be received when there is no specular reflection path. Hence, if additional illumination sources are added which have different angles of incidence, then it is possible to extend the usable range of the sensor. Future work will initially optimize the location and mounting angles of two laser sources for the tracking of uni-directional waves. The eventual aim will be to extend this optimization for the effective tracking of directionally spread waves: aiming to remove steepness limitations (up to established wave steepness limits) for waves propagating over 360^°^. In addition, the overall measurement area should be made larger to maximize the usable data collected by the SPAD detector array. This can be achieved through increasing the illuminated area and can be readily incorporated into the optimization for the location of laser sources. If successful, this will provide the basis for the design of a potentially very powerful wave-measurement system for use in laboratory and ocean settings. A potential arrangement of laser sources to overcome this steepness limitation is depicted in figure 15. The laser sources would be activated in a rapid, time-interleaved, sequence, to obtain full spatial coverage.

Although a truly monostatic arrangement would be ideal from the perspective of parallax, the practical optics requirements are challenging and lateral spacings are required to overcome the steepness limitation. Future work will consider specifically accounting for these geometric effects, which may become significant for large wave amplitudes (relative to the height of the camera system), and may require pixel and surface elevation-dependent correction factors to be applied to the data to improve accuracy, especially as the captured surface area is increased.

## 6. Conclusion

Through experiments, we assess the ability of a SPAD detector array—synchronized with a laser source—to measure surface gravity waves. A range of wave conditions are tested with various frequencies and amplitudes—resulting in a range of wave steepness values (*ka*). The preliminary results are promising and demonstrate that good quality data can be collected up to a limiting value of *ka*. This limit is well predicted by the presented optical model which shows that the data collection capability is highly macropixel dependent: a function of the angles of incidence of light rays for different wave phases. For the presented configuration, a macropixel directly beneath the SPAD detector array and laser is predicted to have a limiting *ka* value of 0.11 which agrees well with the experimental data. However, errors still exist for the lower steepness conditions. It is concluded that the results can be significantly improved by using a higher intensity laser covering a larger area. To increase the range of wave steepness’ where the sensor can successfully collect meaningful data, it is concluded that additional light sources are required which can remove this limitation entirely. This preliminary assessment proves that SPAD detector arrays can be used to measure surface gravity waves, providing real-time measurements at multiple points simultaneously, and has the potential to be a powerful wave measurement device. With some modifications this will enable high-resolution data to be obtained over an entire area of the water surface, without the use of dyes and with low-powered, eye-safe, lasers.

## Data accessibility

Data from the SPAD detector array and corresponding wave gauge data are available at https://doi.org/10.7488/ds/2811.
